# Ubiquitin-specific peptidase 3 induces TPA-mediated leukemia cell differentiation via regulating H2AK119ub

**DOI:** 10.1080/19768354.2019.1661283

**Published:** 2019-09-06

**Authors:** Yun-Cheol Chae, Hyeonsoo Jung, Ji-Young Kim, Dong Ho Lee, Sang-Beom Seo

**Affiliations:** a Department of Life Science, College of Natural Sciences, Chung-Ang University, Seoul, South Korea

**Keywords:** Leukemia cell differentiation, TPA, HL-60 cells, AML, ubiquitin-specific peptidase 3

## Abstract

Acute myeloid leukemia (AML) is the most common type of leukemia in adults. Owing to the chemotherapy associated side effects and toxicity, it is necessary to find a new mechanism, which can identify new potential therapeutic targets at the molecular level. Here, we identified new target genes that are induced during the TPA-induced HL-60 cell differentiation by ChIP-seq and microarray data analysis. Using q-PCR and ChIP assay, we confirmed that the target genes including *USP3*, *USP35*, *TCF4*, and *SGK1* are upregulated during TPA-mediated HL-60 cell differentiation. Levels of USP3, one of the deubiquitinating enzymes (DUBs), increased by TPA treatment, resulting in the reduction of H2AK119ub levels. In addition, we revealed that depletion of USP3 inhibits TPA-mediated leukemia cell differentiation q-PCR and FACS analysis. Taken together, our data indicate that USP3 promotes TPA-mediated leukemia cell differentiation via regulating H2AK119ub levels.

## Introduction

Acute myeloid leukemia (AML) is a malignant disease, which is characterized by altered and reduced production of healthy hematopoietic cells. These alterations inhibit leukemia cell differentiation and induce cell proliferation or accumulation of blast cells in the bone marrow (Fourgeaud et al. 2015; Prada-Arismendy et al. 2017). AML is the most common acute leukemia in adults. However, most of the patients cannot undergo chemotherapy due to its side effects and toxicity. Since the pathophysiological roles of AML are being researched at the cellular and molecular level, it is necessary to identify a new molecular mechanism and optimal treatment strategy for myeloid leukemia.

HL-60 cells, which are human AML cells, have been used as an excellent model for HL-60 cell differentiation by chemotherapeutic agents. HL-60 cells can be induced to differentiate into granulocytes, monocytes, and macrophages by several agents, such as retinoic acid (RA), ascorbic acid (vitamin C), vitamin D, and 12-O-tetradecanoyl phorbol-13-acetate (TPA), respectively (Zylber-Katz and Glazer 1985; Trayner et al. 1998; Yiang et al. 2015). TPA decreased the number of myeloblasts and relieved remissions in some of the patients (Han et al. 1998). However, the mechanism of differentiation by these agents has not been fully understood.

Ubp8, a homologous of ubiquitin-specific peptidase 3 (USP3), has been identified as a functional deubiquitinating enzyme (DUB) that regulated H2B ubiquitination *in vitro* (Henry et al. 2003). However, USP3 has a deubiquitination activity towards H2A and H2B *in vivo*, which causes delayed S phase progression (Nicassio et al. 2007). In addition, USP3 counteracted RNF168 via regulating H2A at K13 and K15 and γH2AX ubiquitination at K118 and K119 in response to DNA damage (Sharma et al. 2014). However, the association between USP3 and TPA-mediated leukemia cell differentiation has not been reported yet.

Here, we identified a new mechanism in leukemia cell differentiation via regulating H2AK119ub by UPS3. The new target genes that were upregulated by TPA-mediated leukemia cell differentiation were identified by analyzing ChIP-seq and microarray data. The knockdown of USP3 inhibited TPA-induced leukemia cell differentiation via regulating H2AK119ub. Therefore, our study indicated that USP3-mediated deubiquitination at H2AK119 may have a therapeutic potential in leukemia cell differentiation.

## Materials and methods

### Cell culture and transient transfection

HEK 293 T cells were grown in Dulbecco’s modified Eagle’s medium (DMEM;Gibco), and HL-60 cells were grown in Roswell Park Memorial Institute (RPMI) 1640 medium (Gibco) containing 10% heat-inactivated fetal bovine serum (Gibco) and 0.05% penicillin–streptomycin (Welgene) at 37°C in 5% CO_2_. 293 T cells were transfected with the DNA constructs using polyethylenimine (PEI) (Polyscience). For differentiation, HL-60 cells were seeded in a 60-mm plate at a concentration of 1 × 10^6^ per mL and treated with 32 nM TPA (Sigma Aldrich) or DMSO (Duchefa).

### RNA interference

This procedure was previously described (Kim et al. 2015). Briefly, DNA oligonucleotides encoding USP3 shRNA (5'–GCTGCCTTTCCACAGCTATAA–3') was subcloned into pLKO.1-puro (Addgene) lentiviral vector according to standard procedures. To produce the viral particles, 293 T cells were co-transfected with plasmids encoding VSV-G, NL-BH, and the shRNAs. Two days after transfection, the media containing the viruses were collected and were used to infect cells in the presence of polybrene (8 μg/ml).

### Antibodies

Antibodies against H3 (05-499), H2AK119ub (05-678; Millipore), and CD11b PE (12-0118-42, eBioscience) were used for western blotting or fluorescence-activated cell sorting (FCAS) analysis.

### Reverse transcription and quantitative PCR (RT-qPCR)

Total RNA was isolated from transfected cells using RNAiso Plus (TaKaRa). After synthesis, cDNA was quantified and subjected to target gene mRNA expression analysis. The following PCR primers were used in this experiment (Table S1). Disassociation curves were performed after each PCR run to ensure that a single product of the appropriate length was amplified. Mean threshold cycle (CT) and standard error values were calculated from individual CT values obtained from triplicate reactions per stage. The normalized mean CT value (^Δ^CT) was estimated by subtracting the mean CT of GAPDH. The value ^ΔΔ^CT was calculated as the difference between control ^Δ^CT and values obtained for each sample. The *n*-fold change in gene expression, when compared to a control and was calculated as 2^−ΔΔ^CT.

### Chromatin immunoprecipitation assay and q-PCR (ChIP-qPCR)

The cells were harvested and cross-linked with 1% formaldehyde. Briefly, 1% formaldehyde was added to the medium for 10 min, followed by the addition of 125 mM glycine for 5 min. HL-60 cells were centrifuged, and the resulting pellets were washed once with 1× phosphate-buffered saline (PBS). The cell pellets were resuspended in sodium dodecyl sulfate (SDS) lysis buffer [1% SDS, 10 mM EDTA, 50 mM Tris-HCl (pH 8.1)]. In addition, the cells were sonicated, and the lysates were subjected to immunoprecipitation (Haferlach et al. 2010) using the antibodies. The immunoprecipitated proteins were eluted and reverse cross-linked. Subsequently, the DNA fragments were purified for PCR amplification. Following this, the DNA fragments were purified and amplified for quantification using each PCR primer pair (Table S1). The mean threshold cycle (CT) and standard error values were calculated from individual CT values from duplicate reactions in each stage.

### Fluorescence-activated cell sorting (FACS) analysis

In this analysis, HL-60 cells were treated with TPA or DMSO for 48 h. The cells were stained with CD11b-PE (BD bioscience) for 30 min. Cells were then subjected to FACS analysis using a BD Accuri™ C6 Plus Flow Cytometer (BD bioscience).

### Histone extraction

To extract histones, cells were resuspended in PBS with 0.5% Triton X-100 and protease inhibitors, and mixtures were subsequently incubated at 4°C for 30 min to lyse the cells. The lysates were centrifuged at 4°C for 10 min at 10,000 *g*, and pellets were resuspended in 0.4 N H_2_SO_4_. Samples were centrifuged at 4°C for 10 min at 16,000 *g*. Pellets were resuspended in 100% trichloroacetic acid for 2 h and centrifuged at 4°C for 10 min at 16,000 *g*. Samples were washed twice with acetone. After removing acetone, histone-containing pellets were collected and eluted in distilled water.

### Statistical analysis

Data are expressed as mean ± standard error of mean (SEM) of three or more independent experiments. Statistical significance (*p* < 0.05) was calculated using Microsoft Excel application. Differences between groups were evaluated by a Student’s *t*-test.

## Results

### Identification of new genes to regulate TPA-mediated HL-60 cell differentiation

**Figure 1.**
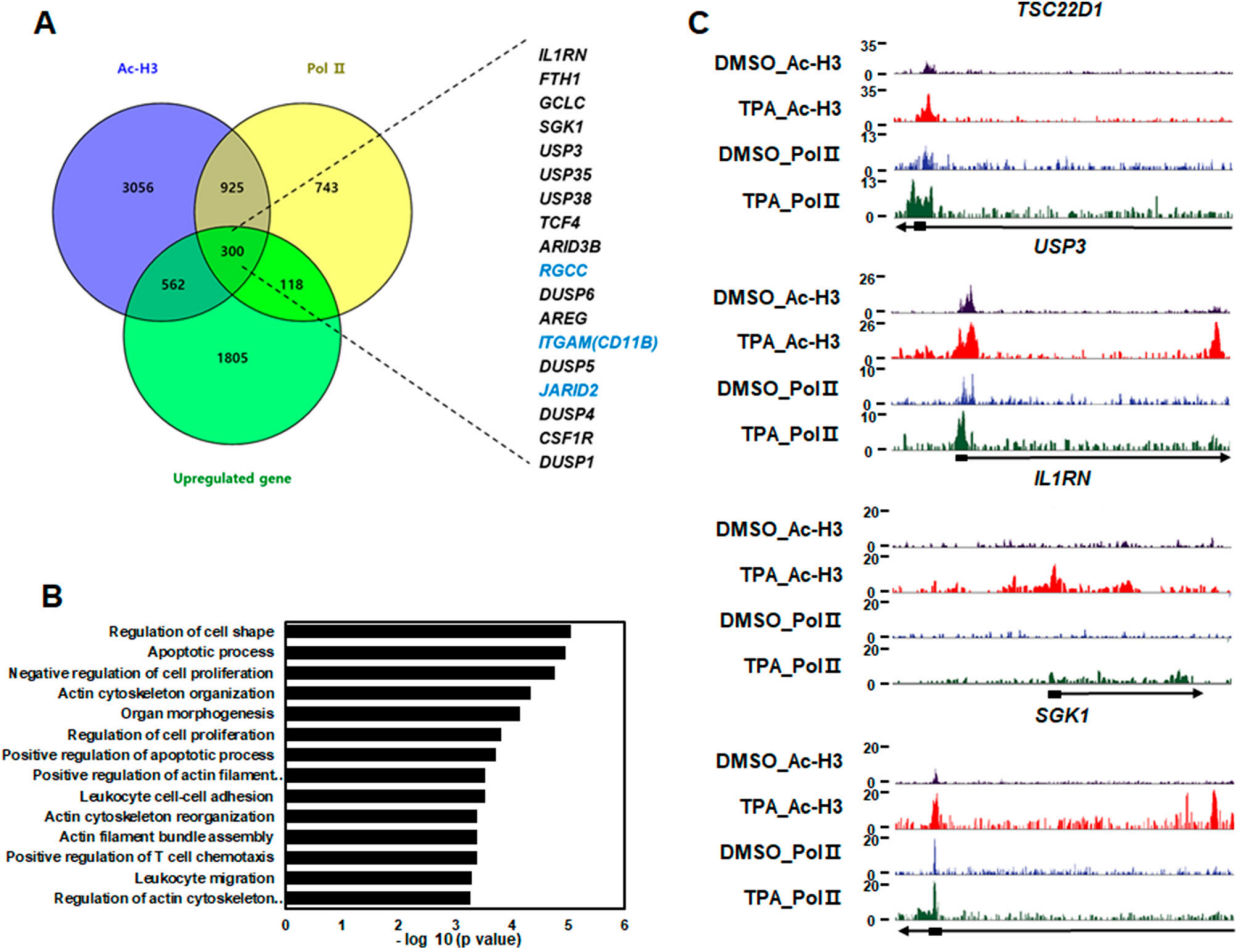
Identification of activated novel genes by TPA treatment using ChIP-seq and microarray data analysis. (A) The Venn diagram showed the overlap between ChIP-seq data (enrichment of Ac-H3 and Pol II) and microarray data analysis (up-regulated genes) during TPA-treated HL-60 cells differentiation. (B) GO terms of overlapping genes in ChIP-seq and microarray data were analyzed in gene ontology consortium. X-axis represents the adjusted *P*-value transformed by -log10, and Y-axis denotes the enriched GO terms. (C) ChIP-seq tracks of AcH3 and RNA Pol II in DMSO or TPA-treated HL-60 cells along the indicated gene loci.

To identify a new mechanism of TPA-mediated leukemia cell differentiation, reanalysis of chromatin immunoprecipitation sequencing (ChIP-seq; GSE110566) and microarray analysis (GSE20476) were performed. The ChIP-seq data were analyzed to reveal RNA Pol II and HDAC2 occupancies from HL-60 cells exposed to TPA or DMSO (control) for 48 h and microarray data were analyzed to identify target genes in control cell and TPA-treated HL-60 cells. Approximately 300 upregulated genes, including *RGCC* and *JARID2* during TPA-mediated leukemia cell differentiation (Figure 1(A)) were obtained. Previous reports demonstrated the inhibitory roles of *JARID2* in leukemia cell differentiation via regulating cyclin D1 (Su et al. 2015). *RGCC* was upregulated during TPA-induced HL-60 cell differentiation via HDAC2 interaction with PAX5 (Jung et al. 2018). We also found *CD11b,* which is known as a differentiation marker, in our analysis, proving the reliability and precisions of the data (Figure 1(A)). Gene ontology (GO) term analysis by gene ontology consortium (http://geneontology.org/page/go-enrichment-analysis) revealed that these genes were related to various cellular process such as regulating actin cytoskeleton, leukocyte migration, and leukocyte cell–cell adhesion, indicating the potential roles of the genes in TPA-induced HL-60 cell differentiation (Figure 1(B)). Next, we chose 10 target genes including *USP3*, *USP35*, *SGK1*, *TCF4*, and *USP38*, which have not been studied well in TPA-treated HL-60, and confirmed that AcH3 and RNA pol II were enriched at the promoter regions of target genes (Figure 1(C)). Our focus was on the 10 target genes, which were upregulated during TPA-mediated HL-60 cell differentiation.

### Induction of target gene mRNA expression during TPA-induced HL-60 cell differentiation

**Figure 2.**
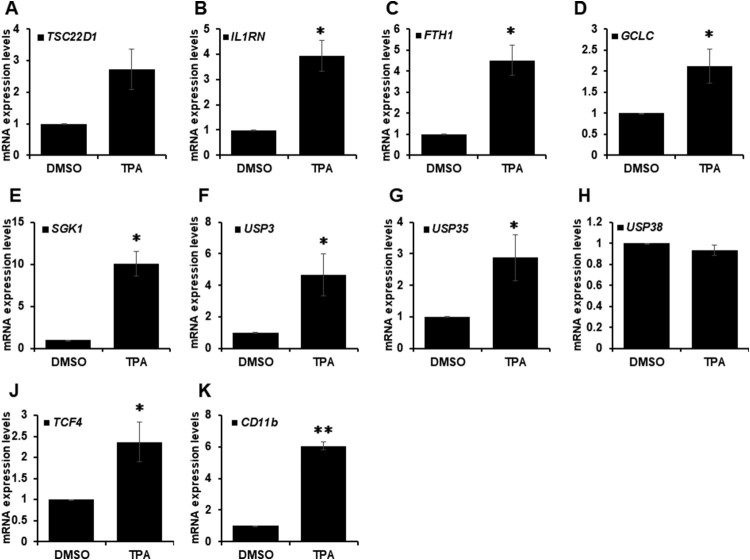
mRNA expression of target genes was validated by RT-qPCR. (A–K) HL-60 cells are analyzed by RT-qPCR to examine the mRNA expression levels of indicated target genes*.* Cells are treated with DMSO or TPA (32 nM) for 48 h. Results are shown as mean ± SEM; *n* = 3. *P < 0.05, **P < 0.01.

To validate the expression level of target genes in TPA-treated HL-60 cells, RT-qPCR in TPA-mediated differentiated HL-60 cells was performed. *CD11b* was significantly upregulated in TPA-treated HL-60 cell, demonstrating that these cells were well-differentiated samples (Figure 2(K)). Most target genes were upregulated during TPA-induced HL-60 cell differentiation including *USP3* and *USP35* (Figure 2(A–J)). However, *USP38* levels did not change in TPA-treated HL-60 cells, indicating that *USP3* and *UPS35* were involved in leukemia cell differentiation. These data coincided with ChIP-seq and microarray data, indicating that these gene levels increased by TPA-mediated HL-60 cell differentiation.

### Validation of ChIP-seq database during HL-60 cell differentiation

**Figure 3.**
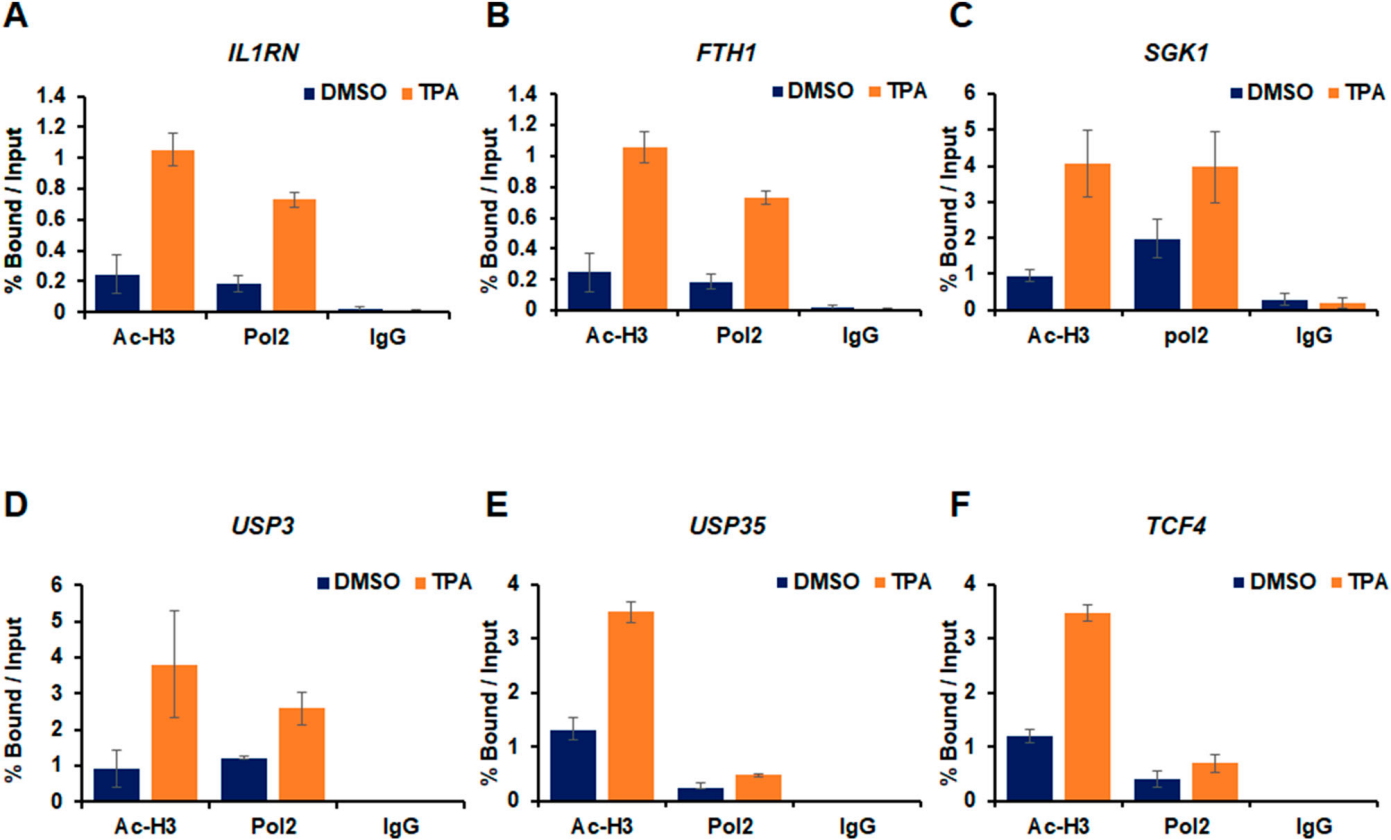
Occupancy of AcH3 and RNA Pol II was confirmed by ChIP assay on target gene loci. (A–F) HL-60 cells are treated with DMSO or TPA (32 nM) for 48 h. (A) The occupancies of AcH3 and RNA Pol II at the promoters of activated genes by TPA are analyzed. The data are normalized by input. These results are shown as mean ± standard deviations (SDs) (*n* = 3).

The occupancies of AcH3 and RNA Pol II on promoter region of target genes in were validated using ChIP-seq data analysis. Among these target genes, we tested the recruitment of AcH3 and Pol II on *IL1RN*, *FTH1*, *SGK1*, *USP3*, *USP35*, and *TCF4* promoter. The genes including *USP3* and *USP35* significantly increased recruitment of Pol II and AcH3 levels in TPA-treated HL-60 cells (Figure 3(A–F)). These data are consistent with previous transcription and ChIP-seq data, demonstrating that these target genes are activated epigenetically during TPA-mediated HL-60 cell differentiation.

### USP regulates leukemia cell differentiation via regulation of H2A119ub

Recently, diverse epigenetic mechanisms have been researched for the therapeutic targets in leukemia. However, the functions of ubiquitin-specific peptidase (USP) in leukemia have not been fully established yet. Since our previous data suggested that USP3 was epigenetically induced by TPA treatment, we focused on the mechanism of USP3 family during TPA-induced HL-60 cell differentiation.

**Figure 4.**
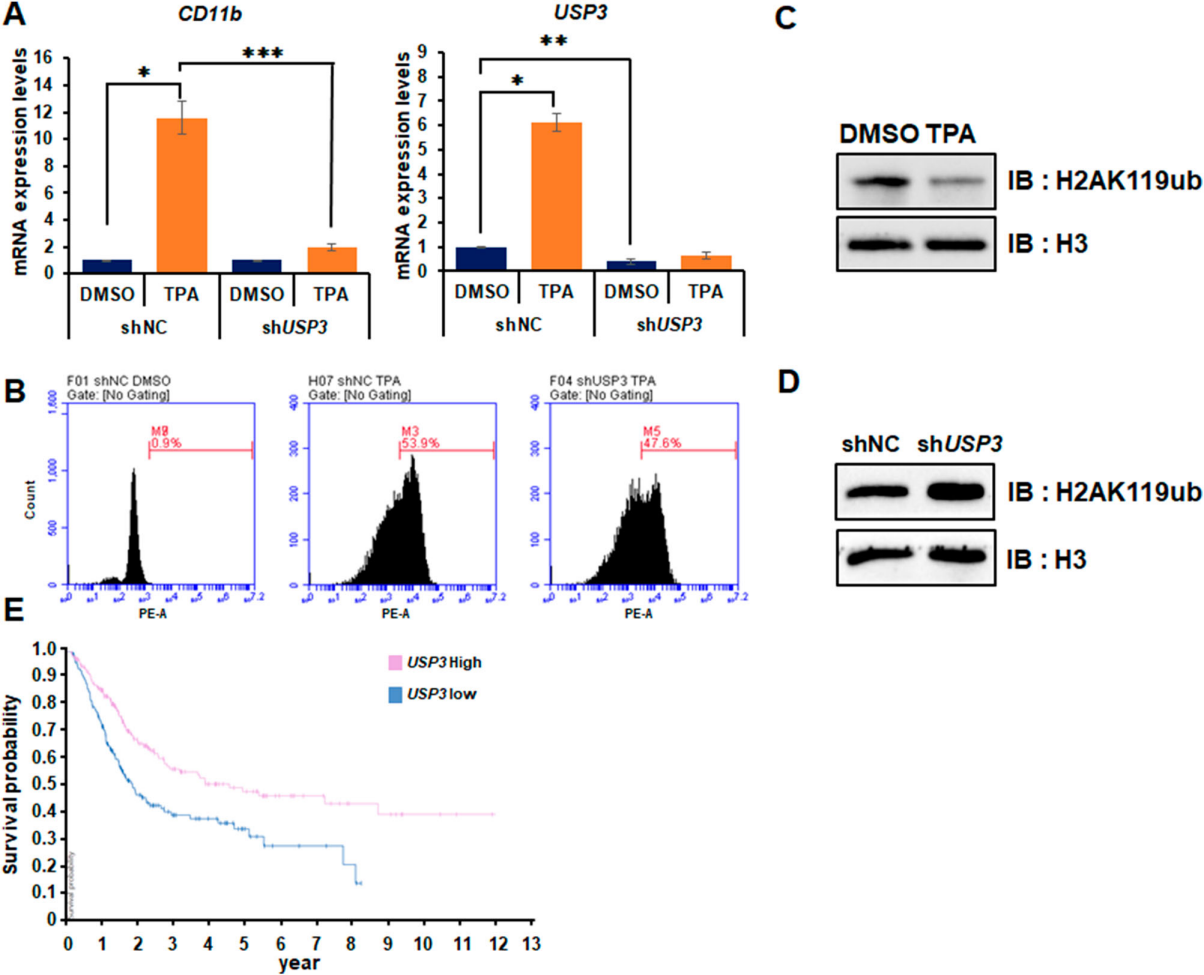
Depletion of USP3 inhibited TPA-mediated HL-60 cell differentiation via regulating H2A119ub. (A) HL-60 cells are analyzed by RT-qPCR to examine the mRNA expression levels of CD11b and USP3*.* Cells are treated with DMSO or TPA (32 nM) for 48 h. Results are represented as mean ± SEM; *n* = 3. *P < 0.05, **P < 0.01, ***P < 0.001. (B) Differentiation of HL-60 cells (CD11b positive) are measured by FACS analysis performed in shNC DMSO, shNC TPA, and shUSP3 TPA. Cells are stained with CD11b-PE for 30 min and analyzed by FACS. (C,D) HL-60 cells are treated with DMSO or TPA for 48 h. Purified histones are resolved by SDS-PAGE and immunoblotted with anti-H3 or anti-H2AK119ub antibodies. (E) USP3 expression levels are showed in comparison with various types of leukemia via the Oncomine database. (F) The probability of survival in urothelial cancer patient is showed in comparison with the level of USP3 expression.

We designed shRNAs of target genes to confirm whether they have an important role in HL-60 cell differentiation and were infected into cells using lentivirus that caused knock-down in target genes. Even though we induced HL-60 cell differentiation by TPA treatment, USP3 depleted HL-60 cells inhibited leukemia cell differentiation, indicating that USP3 promotes leukemia cell differentiation (Figure 4(A)). In addition, the FACS analysis showed that USP3 depleted HL-60 cells also reduced TPA-induced HL-60 cell differentiation (Figure 4(B)). Recently, epigenetic mechanisms on leukemia have been researched as therapeutic targets. However, the functions of USP in leukemia have not been completely understood. Therefore, we tried to reveal the mechanism of leukemia cell differentiation via USP family. USP3 not only regulated ubiquitination of H2A at K13 and 15 but also ubiquitination of **γ**H2AX at K118 and K119 (Sharma et al. 2014). Since H2A also had same conserved K119 residue as **γ**H2AX, we checked the H2AK119ub levels in USP3 depleted 293 T and HL-60 cells. H2AK119ub levels decreased strongly during TPA-mediated leukemia cell differentiation (Figure 4(C)). Moreover, when USP3 was depleted in 293 T and HL-60 cells, H2AK119ub levels reduced significantly (Figure 4(D)). The human protein atlas showed that decreased expression of USP3 has a poor prognosis in urothelial cancer patients thus might act as a tumor suppressor in leukemia and urothelial cancer (Figure 4(E)). Hence, USP3 plays a critical role in TPA-induced HL-60 cell differentiation via regulating H2AK119ub.

## Discussion

Polycomb group (PcG) proteins exist in two types of complexes, polycomb repressive complex 1 (PRC1) and polycomb repressive complex 2 (PRC2). This complex mediates gene silencing through catalysis of histone target residues. PRC1 complex is responsible for H2AK119ub via Ring1B, which has E3 ligase activity (Yuan et al. 2011; Neff et al. 2012) and is associated with leukemogenesis via H2AK119ub. Overexpression of Cbx7, one of the PRC1 subunit, in hematopoietic and progenitor cells promoted leukemia (Klauke et al. 2013). In addition, loss of PRC1 induced leukemia cell differentiation via its enzyme activity towards H2AK119ub showed that these are the two critical factors that maintain undifferentiated state of leukemia cells (Rossi et al. 2016). Therefore, USP3 may counteract to PRC1 complex to regulate cancer development including leukemia via removal H2AK119ub.

USP3 is reported to be associated in cancer development. For example, USP3 activity is essential for hepatocyte growth factor-induced scattering. Depletion of USP3 leads to loss of cell–cell contacts and motility, indicating its role in adjusting cancer cell activity (Buus et al. 2009). USP3 knock out (KO) cells were interrupted to preserve chromosome integrity and USP3 KO mice promote tumor development (Lancini et al. 2014). Moreover, USP3 regulates H2AK119ub levels in **γ**H2AX at K118 and K119 (Sharma et al. 2014). It was found that USP3 decreased H2AK119ub levels in TPA-treated HL-60 cells, resulting in inducing leukemia cell differentiation.

In addition, the human protein atlas suggested that rate of survival of urothelial cancer patients decreased in USP3-low urothelial cancer patients. Oncomine database also showed that level of USP3 decreased in various types of leukemia including AML. These data supported our finding that USP3 might have a tumor suppressor activity.

A new mechanism of leukemia cell differentiation, regulation of H2A119ub by USP3, was demonstrated. Microarray and ChIP-seq data was performed to identify new target genes during TPA-induced HL-60 cell differentiation. However, USP3 depleted cells inhibited TPA-mediated leukemia cell differentiation and were compared with control cells. We also observed that USP3, which was induced during TPA-induced HL-60 cell differentiation, resulted in decrease in H2AK119ub levels. Altogether, USP3 promoted leukemia cell differentiation via regulating H2AK119ub.

## Supplementary Material

Supplemental Material
