# Supplementary material for: Ubiquitin-specific peptidase 3 induces TPA-mediated leukemia cell differentiation via regulating H2AK119ub
Source: Anim Cells Syst (Seoul). 2019 Sep 6;23(5):311–7. doi: 10.1080/19768354.2019.1661283 (PMC6830191; doi:10.1080/19768354.2019.1661283)
Supplement: Supplemental Material [file TACS_A_1661283_SM9147.pdf]

q-PCR primer		
**Gene**	**Forward primer**	**Reverse primer**
**TSC22D1**	TTTAGGTCAGCAGCCTTCCA	CCTGGGGCCATCTGTGTAGA
**IL1RN**	CCGCAGTCACCTAATCACTC	GGCTTGCATCTTGCTGGATTT
**FTH1**	GGCCACTGACAAAAATGACCC	AGAGATATTCCGCCAAGCCAG
**GCLC**	GAGGTCAAACCCAACCCAGT	AAGGTACTGAAGCGAGGGTG
**SGK1**	CTGCAGAAGGACAGGACAAAGC	GACAGGCTCTTCGGTAAACTCGG
**USP3**	AGCAAGATAAAGTTCAGCACACA	ACTTTCTGTACCAGTCCCAGC
**USP35**	CTACTTCCTGTCCCCCGAGA	GTGAGGATGAGGTAGCACGG
**USP38**	TGGAGACCCACCTCTACAGAA	CTGGTGGGTCGTTGTCATCA
**TCF4**	TCTTCCTCCGATGTCCACTTTCCA	CAGGAGGCGTACAGGAAGAGGT
**CD11b**	CGATTCCGTGTTCACCCTGC	TGCCGCTTGAAGAAGCCGAG

ChIP q-PCR primer		
**Gene**	**Forward primer**	**Reverse primer**
**IL1RN**	CGGGTGCTACTTTATGGGCA	TGAGAGTGGAAGGAGCTTACC
**FTH1**	CAGGAAACCCCGACGACTC	CCGACGCGGCTATAAGAGA
**GCLC**	GATCCTGCGCTCCAGGTTTT	TCAACTGCGACCCAATCACC
**SGK1**	CTGCGCGACAGTGAGAAGTG	TCAATGGGGACAGAACCGC
**USP3**	CTGTCTGCCCGAGAGGTTTT	ACTGAACCTATGTGGTGCGG
**USP35**	TGTATGTCGAAGTGATCCTGCTC	ATGTACTAGGTACTGGGTGCGA
**USP38**	CAGGTGTCGGTTCTTAGGCT	ATTTATGGCGCAGGGAGACC
**TCF4**	GGCAATTTTTGGGGGTGGATGT	TGCTGGCGAACAGGGACTT
